# Identifying refugia and corridors under climate change conditions for the Sichuan snub‐nosed monkey (*Rhinopithecus roxellana*) in Hubei Province, China

**DOI:** 10.1002/ece3.4815

**Published:** 2019-02-08

**Authors:** Yu Zhang, Céline Clauzel, Jia Li, Yadong Xue, Yuguang Zhang, Gongsheng Wu, Patrick Giraudoux, Li Li, Diqiang Li

**Affiliations:** ^1^ Chinese Academy of Forestry/Key Laboratory of Biodiversity of National Forestry and Grassland Administration Research Institute of Forest Ecology Environment and Protection Beijing China; ^2^ Key Lab of Hazard Risk Management and Wildlife Management and Ecosystem Health Yunnan University of Finance and Economics Kunming China; ^3^ LADYSS, UMR7533‐CNRS, University Paris Diderot Sorbonne Paris Cité Paris France; ^4^ School of Urban Management and Resource Environment Yunnan University of Finance and Economics Kunming China; ^5^ Chrono‐Environnement, UMR 6249 CNRS University of Bourgogne Franche‐Comté Besançon France

**Keywords:** circuit theory, climate change adaptation, connectivity, golden snub‐nosed monkey, habitat suitability, refuge

## Abstract

Using a case study of an isolated management unit of Sichuan snub‐nosed monkey (*Rhinopithecus roxellana*), we assess the extent that climate change will impact the species’ habitat distribution in the current period and projected into the 2050s. We identify refugia that could maintain the population under climate change and determine dispersal paths for movement of the population to future suitable habitats. Hubei Province, China. We identified climate refugia and potential movements by integrating bioclimatic models with circuit theory and least‐cost model for the current period (1960–1990) and the 2050s (2041–2060). We coupled a maximum entropy algorithm to predict suitable habitat for the current and projected future periods. Suitable habitat areas that were identified during both time periods and that also satisfied home range and dispersal distance conditions were delineated as refugia. We mapped potential movements measured as current flow and linked current and future habitats using least‐cost corridors. Our results indicate up to 1,119 km^2^ of currently suitable habitat within the study range. Based on our projections, a habitat loss of 67.2% due to climate change may occur by the 2050s, resulting in a reduced suitable habitat area of 406 km^2 ^and very little new habitat. The refugia areas amounted to 286 km^2^ and were located in Shennongjia National Park and Badong Natural Reserve. Several connecting corridors between the current and future habitats, which are important for potential movements, were identified. Our assessment of the species predicted a trajectory of habitat loss following anticipated future climate change. We believe conservation efforts should focus on refugia and corridors when planning for future species management. This study will assist conservationists in determining high‐priority regions for effective maintenance of the endangered population under climate change and will encourage increased habitat connectivity.

## 1 INTRODUCTION

Outcomes of climate change challenge the conservation and persistence of many species (Gouveia et al., 2016), especially those considered to be endemic and threatened with higher extinction risks (Lambers, 2015). Climate change can lead to changes in characteristics of movement and species distribution (Lister, Brocki, & Ament, 2015), including range shifts, habitat contractions and expansions and fragmentation (Parmesan, 2006; Struebig et al., 2015). Projections of spatial variations in species distributions and the identification of regions where species could persist and later expand during climate change are essential for targeted conservation efforts (Lambers, 2015; Struebig et al., 2015). Generally, refugia have relatively stable climates and thus facilitate the survival of many taxa under quickly changing environmental conditions (Ashcroft, 2010; Keppel et al., 2015; Li et al., 2016).

The identification of refugia relies on recognition of the current and future distributions of species (Keppel et al., 2015). Habitat suitability assessments based on species distribution modeling (SDM) have been widely used to understand the species response to environmental changes (Gouveia et al., 2016) and to identify refugia (Keppel et al., 2012). Alternatively, climate change velocity can also be used to identify refugia, as these areas characterized by low climate change velocity (Keppel et al., 2015; Sandel et al., 2011). In addition, potential refugia may be recognized by investigating analogues set to current climatic conditions and disturbance distributions (Keppel et al., 2015). However, determining if a species can adjust to shifting climatic conditions by altering its current distribution to locations of climatic refugia or optimal habitat is challenging (Lambers, 2015) because the accessibility of analogue climate habitats is constrained by landscape permeability from anthropogenic modifications and species dispersal capabilities (Littlefield, McRae, Michalak, Lawler, & Carroll, 2017). Thus, maintaining habitat connectivity, the degree to which organisms can move through a landscape (Taylor, Fahrig, Henein, & Merriam, 1993), is the most commonly recommended strategy for conserving species diversity in a changing climate (Heller & Zavaleta, 2009; Nuñez et al., 2013).

Increasing connectivity is critical for species’ ability to track rapidly changing climates and for reconfiguring habitats to facilitate access to more suitable habitats (McGuire, Lawler, McRae, Nuñez, & Theobald, 2016). Previous studies of connectivity analysis are used to identify areas that promote species movements between their current habitats (Brodie et al., 2015; Larue & Nielsen, 2008; Wang et al., 2014). However, this analysis does not consider that suitable areas will shift with climate change; thus, it does not determine key areas of importance under changing climate conditions. Although some papers have addressed the shifting of habitats by modeling connectivity for climate change, for example, Nuñez et al., (2013), Littlefield et al. (2017) and Brost and Beier (2012), the majority of these studies have not considered specific species, but instead addressed “coarse‐filter” conservation that ignores species‐specific traits. Therefore, assessments of the impacts of climate change on connectivity and distribution of specific species are needed, particularly those with high conservation values (Schmitz et al., 2015).

The Sichuan snub‐nosed monkey (*Rhinopithecus roxellana*) is a primate species endemic to China (Li, 2004) and is listed as Endangered by the International Union for Conservation of Nature (Long & Richardson, 2008). *R. roxellana* currently occurs in three isolated temperate montane forest regions in China with a total of approximately 22,000 individuals, comprised of approximately 16,000 individuals in the Sichuan‐Gansu population, approximately 5,500 individuals in the Shanxi population, and approximately 1,000 individuals in the Hubei population (Chang, Luo, et al., 2012). *R. roxellana* has suffered a population decline of more than 50% over the past half‐century (Li, Pan, & Oxnard, 2002). Currently, the major threats to its population are tourism‐related activities and continued habitat loss (Long & Richardson, 2008). Hubei Province is at the easternmost edge of the species distribution and harbors over 1,000 individuals (Liu et al., 2015). This population is considered as a stand‐alone management unit (Chang, Luo, et al., 2012), determined to be a group of conspecific individuals among which the degree of connectivity is sufficiently low and thus should be monitored and managed separately (Palsbøll, Bérubé, & Allendorf, 2007; Taylor & Dizon, 1999). The relatively lower genetic diversity, genetically distinct status and small population size of this population makes it more vulnerable to environmental change than the other two populations (Li et al., 2007; Luo, Pan, Liu, & Li, 2012; Pan et al., 2009). This arboreal species lives in temperate broadleaf and coniferous forests (Chang, Liu, Yang, Li, & Vigilant, 2012), and the impact of climate change on vegetation is expected to decrease the availability of its suitable habitat (Luo et al., 2015; Xiang et al., 2011). These predicted range reductions will force the Hubei population monkeys to migrate to higher elevations over time (Lou et al., 2015). In addition, there is little research identifying the refugia of *R. roxellana* and how it may disperse from its currently suitable habitats to future habitats. Identifying the extent to which the *R. roxellana* could be affected by climate change and the dispersal paths between its current and future habitats are therefore important for effective conservation management.

Here, we built a *R. roxellana* distribution model based on monkey occurrences associated with bioclimatic and environmental variables. We aimed to (a) assess the extent that climate change will impact the *R. roxellana* habitat distribution from the current period to the 2050s, (b) identify refugia locations that could maintain the population under climate change and dispersal paths that could facilitate the movement of the population to suitable future habitats, and (c) provide suggestions for the conservation of *R. roxellana* in Hubei Province under environmental change.

## 2 METHODS

### 2.1 Study area and population of *R. roxellana*

**Figure 1:**
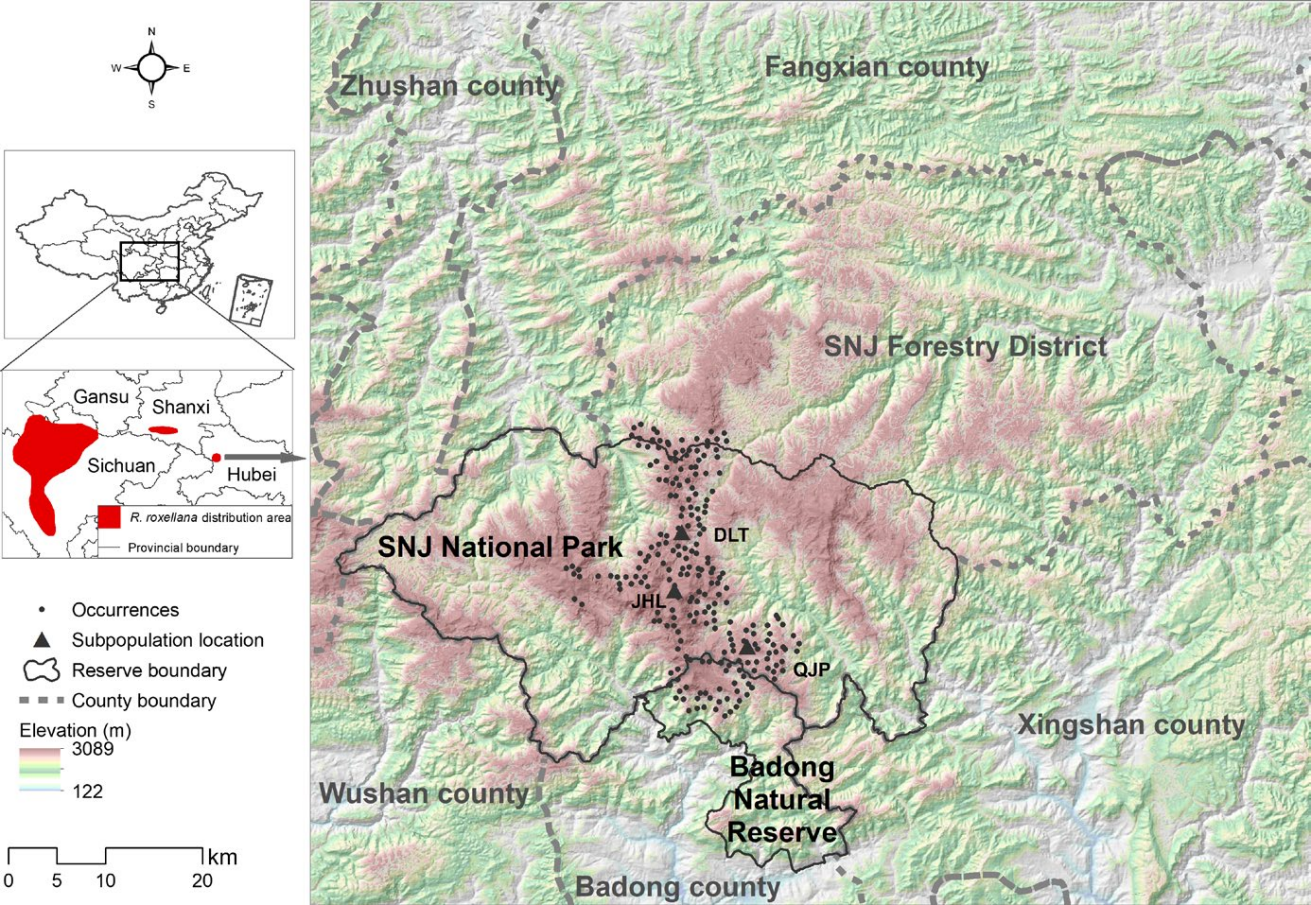
Map of the study area in Hubei Province and the occurrences and locations of subpopulations of *Rhinopithecus roxellana* (DLT, JHL, and QJP refer to the Dalongtan, Jinhoulin, and Qianjiaping subpopulations, respectively). The population in Hubei Province was found in the Shennongjia (SNJ) National Park, Shennongjia Forestry District, and Badong Natural Reserve

This study was conducted at the main distribution area of *R. roxellana* in Hubei Province (Figure 1). The study area was expanded appropriately to cover larger areas in the surrounding region, including the entire Shennongjia (SNJ) Forestry District, the Badong Nature Reserve, and other areas within the rectangular range in an effort to prevent omissions of *R. roxellana *likely habitat. The SNJ National Park is located in the SNJ Forestry District; however, this paper uses the term “SNJ Forestry District” to refer to the area not in the SNJ National Park. This paper mainly involves three areas, that is, The SNJ Forestry District, SNJ National Park, and Badong Nature Reserve (Figure 1). There are three monkey subpopulations, Dalongtan (DLT), Jinhoulin (JHL), and Qianjiaping (QJP), that inhabit the SNJ National Park (Yang, Liao, Yu, & Yao, 2008). A substantial area of the SNJ National Park suffered deforestation between the 1950s and the early 1980s (Zhu, 1992). In recent years, tourism at the SNJ has developed rapidly (Xiang et al., 2011), resulting in human disturbances near scenic locations and habitat fragmentation caused by the increased traffic. Human disturbances in the SNJ Forestry District are mainly concentrated in the spring, when villagers collect Chinese medicinal herbs.

### 2.2 Occurrence records

The occurrence records of *R. roxellana* (*N* = 1,225) comprised data from standard monitoring activities by the staff of the SNJ National Park from May to December 2013, camera‐trap monitoring data and the published literature (Luo et al., 2015; Su et al., 2004). To diminish the impact of spatial autocorrelation, we filtered multiple records by randomly selecting one record in a 1 km × 1 km grid cell. A total of 234 occurrence records were used in this study (Figure 1).

### 2.3 Environmental variables

We obtained a set of climatic variables from the WorldClim database (http://www.worldclim.org) at a 30” resolution to define the species distribution models for current climate (average for 1960–1990) and future climatic (average for 2041–2060) scenarios. The projection was based on the general circulation models of HadGEM_2_‐AO of the Fifth Assessment Report of the International Panel of Climate Change. The simulation used HadGEM_2_‐AO which performed well in the East Asia region (Baek et al., 2013). The scenario was described in terms of the Representative Concentration Pathways 4.5, which predict an average global temperature increase of 0.9–2.0°C by the 2050s (UNFCCC, 2015). The time horizon of the 2050s was selected because it is a period far enough in the future for significant climate changes to have occurred (Young et al., 2012).

Other environmental variables (Supporting Information Table S1) used to construct the *R. roxellana* distribution models included density of rivers, roads and settlements and vegetation types, which were obtained from a 1:1,000,000 map of China (National Geomatics Center of China). The elevation was derived from a digital elevation model with a resolution of 30″ from the WorldClim database. Non‐climate variables are not available for the 2050s, and vegetation changes occur slowly, so we kept these variables static in our projections (Li, Liu, Xue, Zhang, & Li, 2017).

All variables were resampled at a resolution of 1 km × 1 km and put into the same projection using ArcGIS 10.1 (ESRI Inc., Redlands, CA, USA). To reduce multicollinearity, a reduced version was produced by eliminating Pearson's correlation coefficients of |*r*| > 0.8 (Cord, Klein, Mora, & Dech, 2014; Supporting Information Table S2), leaving seven variables to construct the *R. roxellana* distribution model (Temperature Seasonality (Bio4), Min Temperature of Coldest Month (Bio6), Precipitation Seasonality (Bio15), Settlement density, Road density, River density, and Vegetation type; Table S1).

### 2.4 Species distribution model

We employed the maximum entropy algorithm (MaxEnt 3.3.3k), one of the best performing approaches in modeling species distribution, with presence‐only data (Elith & Yates, 2015) to construct the habitat suitability for *R. roxellana* under the current day scenario and then projected the spatial information into the 2050s. We used the default settings for the MaxEnt model (Phillips, Anderson, & Schapire, 2006), with the exception of dividing the percentage at 75% of the occurrence data into training set for model construction and the percentage at 25% into a random test set for evaluation of model performance. We conducted a subsampling procedure with 15 replicates (Khatchikian, Sangermano, Kendell, & Livdahl, 2011).

To evaluate the model performance, we used the threshold‐independent area under the receiver operating characteristic curve (AUC) with value ranges from 0 to 1. AUC values close to 1 indicate perfect model agreement (Phillips et al., 2006). Variable importance was estimated by the permutation importance method (Searcy & Shaffer, 2016). The logistic results of the MaxEnt model were considered to represent the probabilities of species occurrence (Phillips & Dudík, 2008). We then reclassified the results into presence and absence using the average of the maximum training sensitivity plus specificity (Liu, Berry, Dawson, & Pearson, 2005; Songer, Delion, Biggs, & Huang, 2012). Areas with probability values above the threshold were regarded as suitable habitats.

### 2.5 Assessing habitat vulnerability and climate refugia

We assessed the impact of climate change on *R. roxellana* based on the suitable habitat changes between the current period and the 2050s. The aim was to identify vulnerable habitats, that is, current suitable habitat that will be lost by 2050, and climatic refugia, that is, areas where suitable habitat was present in the current period and in the 2050s projection. The home range of the species is 18.3 km^2^, of which 7.4 km^2^ is considered core area (Tan, Guo, & Li, 2007). The daily path length varies from 0.75 to 5 km with a mean of 2.1 km (Tan et al., 2007). *R. roxellana* has a male‐based dispersal system (Chang et al., 2014; Huang et al., 2017). We parameterized our models of refugia according to those values; for example, patch areas had to be >7.4 km^2^, and the distance to the nearest patch had to be <2.1 km and located in a protected area for more effective management and protection. Three indicators were employed to assess the habitat vulnerability, including the percentage of suitable habitat area change (AC), the percentage of currently suitable area that was lost by the 2050s (SH_L_), and the percent increase of future suitable area by the 2050s (SH_I_). Indicators were calculated as follows:

$$\text{AC}=\frac{A_{\text{F}}-A_{\text{C}}}{A_{\text{C}}}\times 100\%$$

$${\text{SH}}_{\text{L}}=\frac{A_{\text{C}}-A_{\text{FC}}}{A_{\text{C}}}\times 100\%$$

$${\text{SH}}_{\text{I}}=\frac{A_{\text{F}}-A_{\text{FC}}}{A_{\text{C}}}\times 100\%$$

where *A*_F_ is the area of the projected suitable habitat for *R. roxellana* under the 2050s climatic scenario; *A*_C_ is the area of the modeled current suitable habitat; *A*_FC_ is the constant area of the suitable habitat in both the current period and the 2050s (Irina, Flemming, Jenschristian, & Carsten, 2007; Li et al., 2017).

The difference in the average elevation of the suitable habitat between the time periods was examined using a Mann–Whitney *U* test. Statistical analyses were carried out using SPSS 19.0 software.

### 2.6 Habitat connectivity analysis

We used Circuitscape v4.0 (McRae, Shah, & Mohapatra, 2013) to quantify potential species movement between suitable habitat patches in the current period and the 2050s. The Circuitscape model connectivity was based on the circuit theory, predicting the movement patterns of random walkers between source and target cells across a landscape. High densities of current flow indicate important movement between habitat patches (McRae, Dickson, Keitt, & Shah, 2008). We ran Circuitscape using the pairwise mode for the current period and the 2050s. The suitable habitat patches in each period were treated as nodes (the source and target). We used the inverse of the logistic output from our MaxEnt model as a measure of movement resistance for *R. roxellana* and rescaled from 1 to 100 to construct a resistance layer (Li et al., 2016). For cells with a value larger than the threshold (maximum training sensitivity plus specificity was given by the MaxEnt model), resistance was set to 1. For cells with a value smaller than the threshold, resistance was set to (threshold ‐ “value”) × 100/threshold. We mapped the current flow between suitable habitats for two states (current and the future climate scenario) to visualize habitat connectivity.

We linked suitable habitats in the 2050s scenario to model potential dispersal paths from current to future habitats. We mapped the least‐cost path and least‐cost corridors between suitable habitats whose distance to the nearest patch was <2.1 km. The resistance layer identical to the input used in Circuitscape. The least‐cost path and corridors were implemented in LinkageMapper (McRae & Kavanagh, 2011), and truncated at a cost distance of 200,000 cost unit for visualization.

## 3 RESULTS

### 3.1 Habitat suitability model

The MaxEnt model for *R. roxellana* provided satisfactory results, with an AUC value of 0.955 (±0.005). Temperature seasonality (bio04, 57.8%, Supporting Information Table S1) contributed the most to the model, according to the permutation importance, followed by the precipitation seasonality (bio15, 21.9%), settlement density (10.4%), vegetation type (4.6%), road density (4.1%), river density (0.7%), and min temperature of the coldest month (bio06, 0.6%). The average threshold value for the measure of suitable habitat was 0.185.

### 3.2 Habitat suitability for the current period and the 2050s

**Figure 2:**
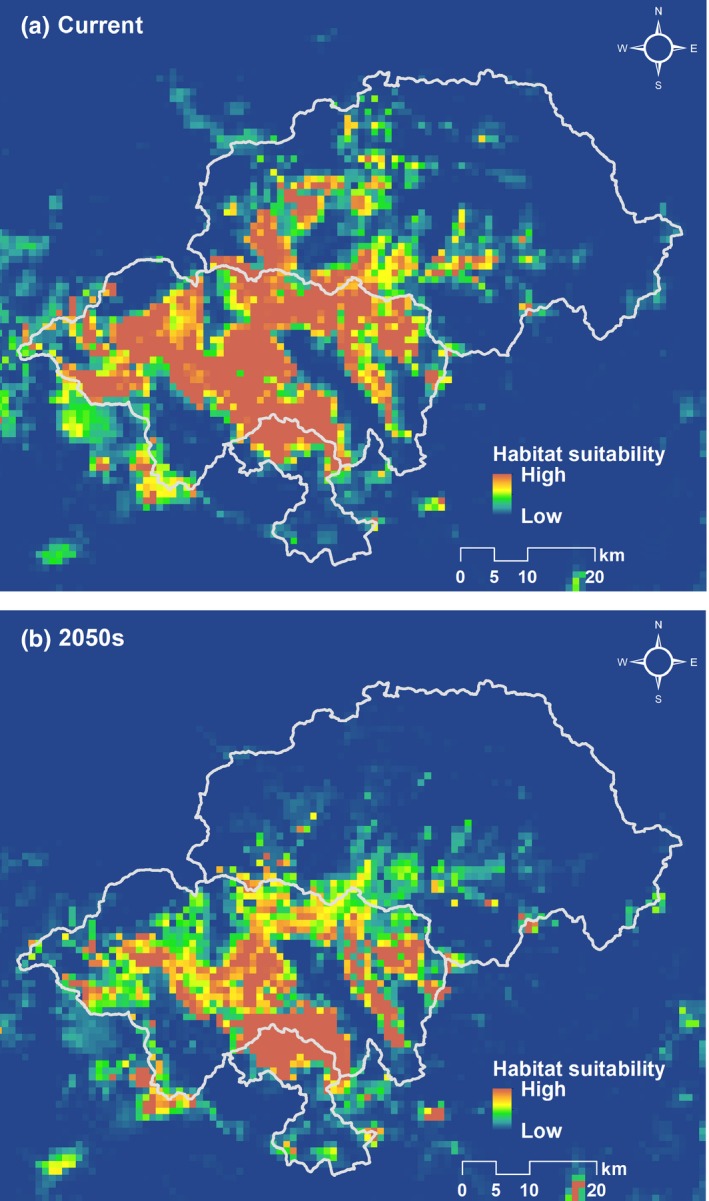
Habitat suitability for *Rhinopithecus roxellana *in Hubei Province. (a) depicts the models under the current climate. (b) is the projection of habitat suitability by the 2050s

For the current period, the area with a habitat suitability value higher than 0.185 was 1,119 km^2^, predominately concentrated in SNJ National Park (722 km^2^), the southwest region of the SNJ Forestry District (247 km^2^), and the northern region of the Badong Nature Reserve (61 km^2^) (Figure 2a). In the 2050s, the suitable habitat area was dramatically reduced to 406 km^2^, representing a decrease of −63.7%. The most suitable habitat retreated to SNJ National Park (293 km^2^, AC = −59.4%) and the northern region of Badong Nature Reserve (60 km^2^, AC = −1.6%). The SNJ Forestry District lost the majority of its suitable habitat, with only 11 km^2^ of existing suitable habitat area remaining in the 2050s (AC = −95.5%) (Table 1, Figure 2b).

**Table 1:** Estimates of the suitable habitat area (km^2^) for *Rhinopithecus roxellana* in Hubei Province in the current period and the 2050s, with the percentage change values

	*A*_C_ (km^2^)	*A*_F_ (km^2^)	*A*_FC_ (km^2^)	AC (%)	SH_L_ (%)	SH_I_ (%)
Study area	1,119	406	367	−63.7	67.2	3.5
SNJ Forestry District	247	11	10	−95.5	96.0	0.4
SNJ National Park	722	293	273	−59.4	62.2	2.8
Badong Nature Reserve	61	60	54	−1.6	11.5	9.8

Notes

*A*_C_ is the area of the modeled current suitable habitat, *A*_F_ is the area of the projected suitable habitat under the climate scenario of the 2050s, and *A*_FC _is the constant area of suitable habitat both in the current and the future (2050s) periods. AC, SH_L_, and SH_I_ refer to the percentage of suitable habitat area change, currently suitable area that was lost by 2050s and increased in future suitable area by the 2050s, respectively.

Suitable habitat scarcely increased in the 2050s (SH_I_ = 3.5%, Table 1, Supporting Information Figure S1). Most of the habitat was shown to be vulnerable to climate change (SH_L_ = 67.2%). Among the main areas, the habitat of the Badong Nature Reserve had the lowest vulnerability to climate change (SH_L_ = 9.8%), followed by the habitat of the SNJ National Park (SH_L_ = 62.2%) and the SNJ Forestry District (SH_L_ = 96.0%).

**Figure 3:**
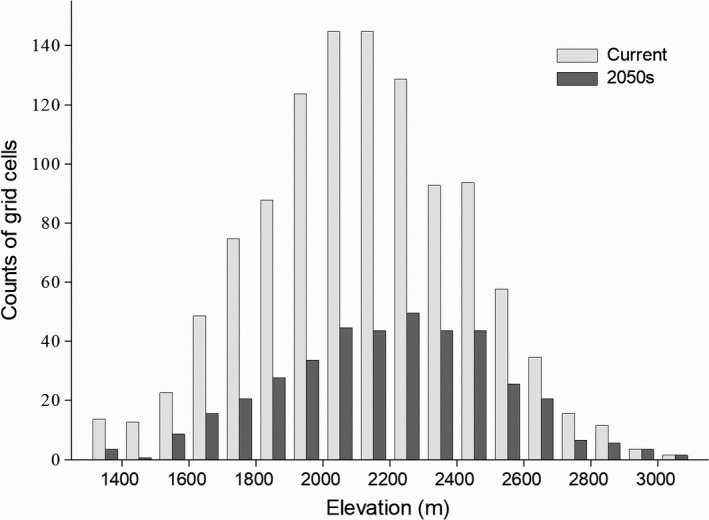
Impacts of climate change on the potential distribution of *Rhinopithecus roxellana* in Hubei Province over elevation. The *y*‐axis represents the frequencies of suitable habitat cell numbers, with 100 m elevation intervals, for the current (light gray) period and the 2050s (dark gray)

Climate change would result in a shift of the suitable habitat of *R. roxellana* to higher elevations (Figure 3). The mean elevation of the suitable habitat in the 2050s was projected to be 2,183.66 ± 325.35 m, which is significantly higher (*Z* = −3.554, *p* = 0.000) than the elevation of the currently suitable habitat (2,118.90 ± 315.94 m).

### 3.3 Climate refugia and potential movement under climate change

**Figure 4:**
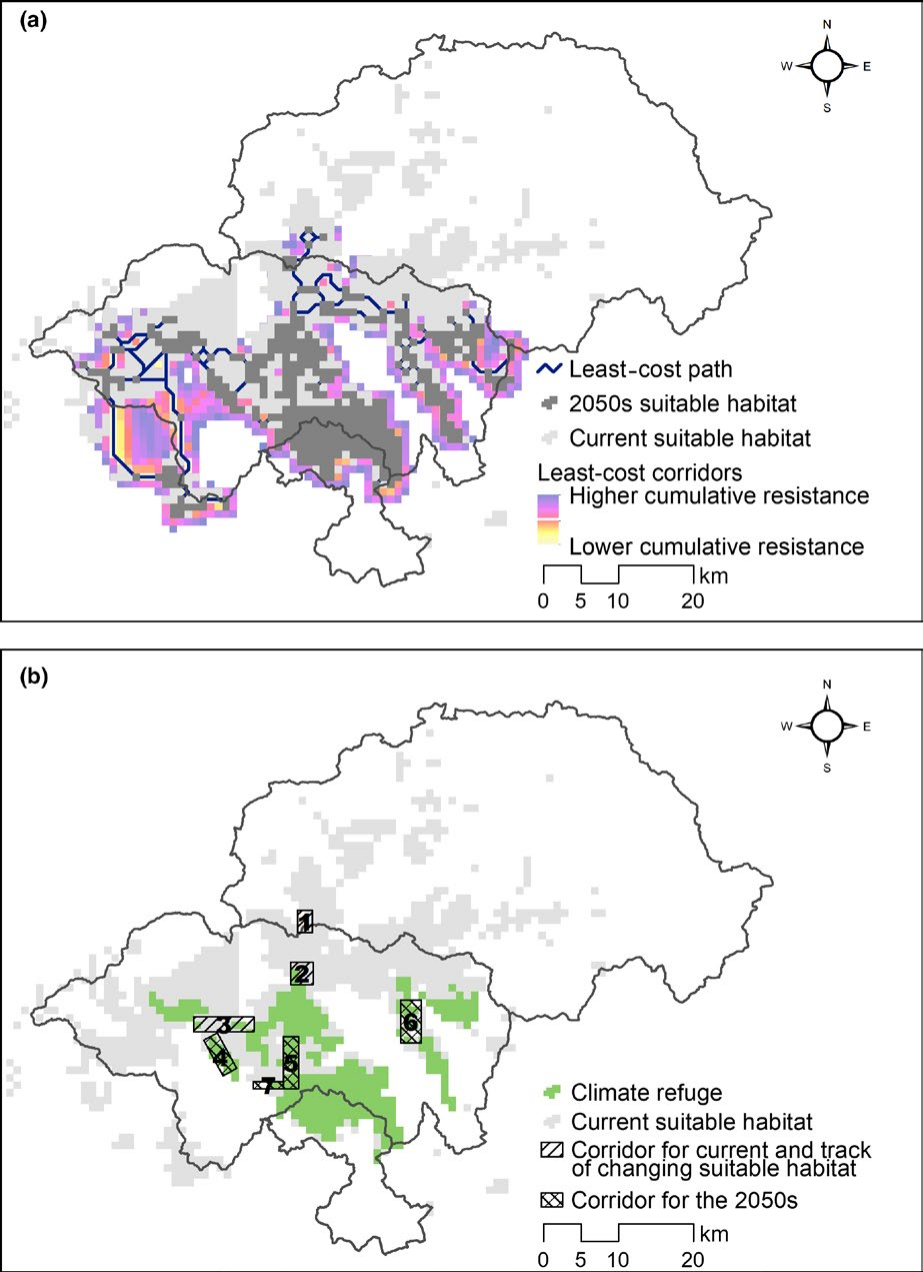
(a) The least‐cost path and corridors between current and future suitable habitats, least‐cost corridors truncated at cost distance of 200,000 cost units. (b) Climate refugia and corridors where priority conservation efforts could be focused. The potential corridor areas numbered 1, 2, and 3 are the more critical areas for current movements and tracking climate change. The corridor areas numbered 4, 5, 6, and 7 are more critical under future conditions

By intersecting areas suitable for *R. roxellana* in the current and future scenarios and filtering areas according to species‐specific parameters, a total area of 286 km^2^ was identified as climate refugia (Figure 4b). In accordance with the parameters, the patch areas had to be >7.4 km^2^, and the distance to the nearest patch had to be <2.1 km and located in a protected area.

**Figure 5:**
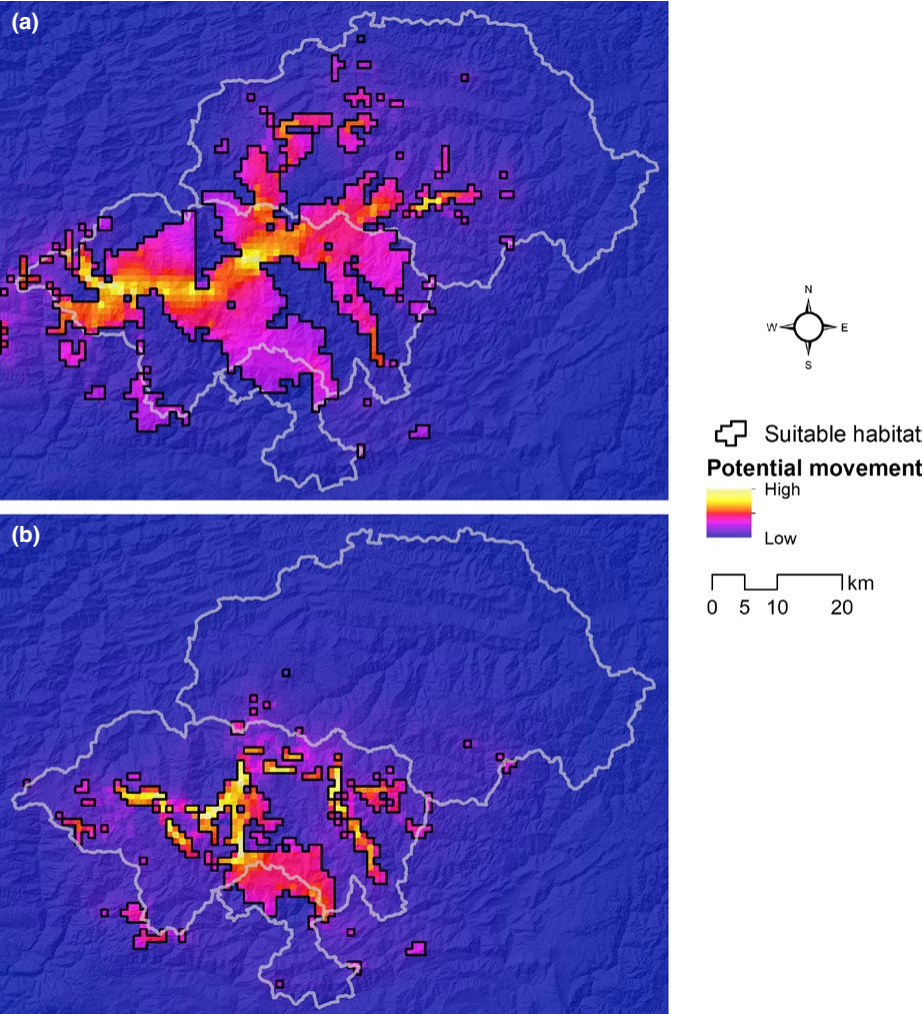
Potential *Rhinopithecus roxellana* movements in Hubei Province, based on circuit theory, between suitable habitats of (a) the current climate and (b) the climate scenario of the 2050s. The color ramp reflects the absolute potential movement values for each situation. Thus, colors are not directly comparable, and the results should be interpreted regarding the relative importance of the potential movement area

The model of circuit theory highlighted “pinch points” of high level movement. In the current situation, several areas exhibited potentially high current flow; therefore, they likely indicate critical pathways for possible movement across suitable habitats (Figure 5a). As habitats shrink and narrow under the conditions modeled for the 2050s, movement will be facilitated by narrowed habitats and severely restricted (Figure 5b). From the least‐cost path and least‐cost corridors, we modeled linkages between current and future habitats (Figure 4a). These linkages emphasized routeways in which *R. roxellana *can navigate from current to future habitats.

## 4 DISCUSSION

In this paper, we assessed the impact of climate change on the *R. roxellana* habitat range and identified refugia and corridors under climate change conditions. Our assessment of the species predicted a trajectory of habitat loss following anticipated future climate change. Additionally, we identified strategic areas that should be prioritized during species preservation efforts. Our analysis provides a perspective to evaluate the impact of climate change on habitat connectivity for a species of conservation concern, and proposes targeted actions that consider species characteristics.

### 4.1 Projected effects of climate change

There have been few published articles about the effects of the changing climate on the *Rhinopithecus* genus to date. A case study about *Rhinopithecus bieti, *which used climate and socio‐economic scenarios to model land cover changes until 2050, predicted that the area of suitable habitats will be reduced by 15% by 2050 (Li, Wu, Xue, He, & Giraudoux, 2011). The potential distribution area of *Rhinopithecus avunculus* will be 20% smaller in 2020 than it is under current climate conditions (Van, Manh, & Hoang, 2010). According to our results, *R. roxellana* in Hubei will be the most seriously impacted species of the *Rhinopithecus* genus, with a habitat decrease of 67.2% and a meager 3.5% addition of newly formed habitat by the 2050s. This suggests that the effects of climate change will greatly exacerbate the vulnerability of *R. roxellana*. These results are consistent with the research of Luo et al. (2015), which predicted a habitat reduction of almost 70% by 2050 in the SNJ area and an increase in the mean elevation of suitable habitat. Compared with Luo's analysis, which omitted the Badong Nature Reserve, we considered the entire distribution area of the Hubei population. Among the three subpopulations, the DLT and JHL subpopulations may be forced to recolonize southward due to substantial habitat loss, likely resulting in intraspecific resource competition that will cause population decline of *R. roxellana*.

### 4.2 Refugia and potential movement under climate change

According to the ecological requirements of the species, we further refined the area of refugia that can sustain subpopulations and the corridors that can serve as priority sanctuaries. Such refugia were primarily located in the SNJ National Park and the Badong Natural Reserve, which suggests the high potential of these regions for use in preserving the population under climate change (Figure 4). Preservation efforts between the two protected areas should focus on building trans‐boundary cooperation systems to ensure habitat connectivity.

Under climate change conditions, *R. roxellana *will be forced to shift their range away from the current habitat areas that will no longer be suitable and arrive at future habitats. The dispersal path of the least‐cost model can improve movement into the 2050s’ optimal habitat (Figure 4a). Moreover, the map connecting the suitable habitats under the current and future scenarios using circuit theory highlights different corridor types that are important for potential movements and emphasizes that corridors are likely to vary in importance with time (Figure 5). The potential corridor areas numbered one, two, and three were the more vital areas for current movements and tracking climate change. Corridors four, five, six, and seven will become important under future conditions. Thus, a staged process of conservation actions could be implemented as follows: Conservation actions could be immediately focused on corridor areas 1–3 through assessment of habitat quality and restoration of degraded habitats; the future corridor areas 4–7 should also be protected to ensure they remain ecologically intact for their future use. Rehabilitation of unsuitable habitats could be taken into consideration around these areas. Priority conservation efforts should be focused on these strategic areas. Likewise, long‐term efforts are also needed to monitor not only habitat quality, including its phenology, food availability, and plant community dynamics, but also the status and movement trends of each subpopulation.

### 4.3 Implication for species conservation

Because climate change is anticipated to alter both the movements and distributions of species, there is a need to take climate change into account during the promotion of adaptive conservation strategies (Schmitz et al., 2015). Investigating connectivity without considering shifts in future habitats would omit areas that are crucial for species movement from current to future habitats. Understanding the connection from current habitats to future habitats can allow for prediction of significant areas necessary for the climate‐induced movement and where interventions for conservation efforts could be focused. For example, Littlefield et al. (2017) identified key areas likely to facilitate climate‐induced species movement across western North America. For habitat reduction at the small study scale, like that investigated in this study, the changes in suitable habitat locations also influence the pathways that animals need to track climate change and reach emerging suitable habitats. This also highlights the importance of protecting the current habitat for such situations. The prediction of the potential movement between future habitats will allow for advanced preparation against the negative impacts of climate change.

Connectivity analysis that considers climate change typically focuses on two scales—regional and individual species. The regional scale takes several species into account simultaneously; therefore, the ecological niche needs of each species, such as specific climatic condition, elevations, slopes, and special vegetation types, cannot be considered in detail. Instead, it uses a coarsely approximate climatic condition to determine current habitat distribution and to predict future conditions. For example, Littlefield et al. (2017) used the multivariate similarity of climates through a principal component analysis to identify climate analogue habitats. The analysis based on the individual species scale, as a refinement of the analysis based on the regional scale, usually requires a species distribution model to simulate future habitats, which can focus on specific requirements. For example, Kang, Minor, Lee, and Park (2016) used a species‐based MaxEnt model to valuate changes in the extent and connectivity of castor aralia (*Kalopanax septemlobus*) habitat, taking bioclimatic and topographic variables into account. The individual species scale analysis can make a complete corridor prediction involving species dispersal capability.

Identifying resistance surface is challenging in connectivity analysis (Milanesi et al., 2017). Ideally, resistance values would be parameterized with empirical data, but because of a shortage of such information, expert knowledge is often used (Stevenson‐Holt, Watts, Bellamy, Nevin, & Ramsey, 2014). However, the use of expert opinion is seen as subjective, human‐centric and unreliable (Puyravaud, Cushman, Davidar, & Madappa, 2017).Thus, the resistance surface converted from the habitat suitability index is superior to the subjective method of expert opinion to some extent. It takes into account a variety of variables affecting the distribution of species, and facilitates the rapid assessment of the connectivity of specific species.

### 4.4 Contributions and limits of the method

Our model identified climate‐induced shifts in the habitat distribution of *R. roxellana* and determined the most effective regions enabling *R. roxellana *to shift its distribution range. However, we made several simplifying assumptions. First, our approach used a relatively coarse resolution of climate data and did not take fine‐scale characteristics into account, which might be central to long‐term survival (Struebig et al., 2015) and provide climate microrefugia for *R. roxellana*. We also used a static map of vegetation and human disturbances, because changes in the vegetation community lag in response to climate change (Barbet‐Massin, Thuiller, & Jiguet, 2012), and the main distribution area was in protected areas where construction projects need strict approval. However, we do not know precisely what kind of changes in land use under climate change might occur with an increasing human population and global economy shifts. Here, we assume that the area will be relatively protected. In addition, our analysis was a species‐specific approach that cannot be directly extended to other species, although the preservation of* R. roxellana* could benefit other sympatric animal species. Nonetheless, we note that *R. roxellana *in Hubei Province is a stand‐alone management unit (Chang, Luo, et al., 2012), and that use a relatively small spatial extent was valuable for comparing the effects of climate change.

The identification of refugia areas and analysis of climate connectivity will allow conservationists to determine the most effective regions for maintaining a population of *R. roxellana* and increasing habitat connectivity, in the context of the climate changes that are predicted for this century.

## CONFLICT OF INTEREST

None declared.

## AUTHOR CONTRIBUTIONS

Yu Zhang and Diqiang Li conceived and designed the work. Yadong Xue, Yuguang Zhang, and Gongsheng Wu collected the data. Yu Zhang, Jia Li analyzed and interpreted the data. Yu Zhang drafted the article. Celine Clauzel, Patrick Giraudoux, and Li Li critically revised the article. Yu Zhang, Céline Clauzel, Jia Li, Yadong Xue, Yuguang Zhang, Gongsheng Wu, Patrick Giraudoux, Li Li, and Diqiang Li gave final approval of the version to be published.

## Data Availability

Climate data and MaxEnt input files for this study will be available at the Dryad after the paper publishing.
