# Supplementary material for: Identifying refugia and corridors under climate change conditions for the Sichuan snub‐nosed monkey (Rhinopithecus roxellana) in Hubei Province, China
Source: Ecol Evol. 2019 Feb 8;9(4):1680–90. doi: 10.1002/ece3.4815 (PMC6392490; doi:10.1002/ece3.4815)
Supplement: Supplementary file 1 [file ECE3-9-1680-s001.docx]

Table S1 Environmental variables used to model the suitable habitat for *R. roxellana* and their relative contributions to the MaxEnt model with permutation importance.

| Code | Environmental variables | Unit | Permutation importance |
| --- | --- | --- | --- |
| Bio1 | Annual Mean Temperature | °C |  |
| Bio2 | Mean Diurnal Range (Mean of monthly (max temp–min temp)) | °C |  |
| Bio3 | Isothermality (Bio2/Bio7) (×100) | – |  |
| **Bio4** | **Temperature Seasonality (standard deviation *100)** |  | **57.8** |
| Bio5 | Max Temperature of Warmest Month | °C |  |
| **Bio6** | **Min Temperature of Coldest Month** | **°C** | **0.6** |
| Bio7 | Temperature Annual Range (Bio5–Bio6) | °C |  |
| Bio8 | Mean Temperature of Wettest Quarter | °C |  |
| Bio9 | Mean Temperature of Driest Quarter | °C |  |
| Bio10 | Mean Temperature of Warmest Quarter | °C |  |
| Bio11 | Mean Temperature of Coldest Quarter | °C |  |
| Bio12 | Annual Precipitation | mm |  |
| Bio13 | Precipitation of Wettest Month | mm |  |
| Bio14 | Precipitation of Driest Month | mm |  |
| **Bio15** | **Precipitation Seasonality (Coefficient of Variation)** |  | **21.9** |
| Bio16 | Precipitation of Wettest Quarter | mm |  |
| Bio17 | Precipitation of Driest Quarter | mm |  |
| Bio18 | Precipitation of Warmest Quarter | mm |  |
| Bio19 | Precipitation of Coldest Quarter | mm |  |
| **Settledensity** | **Settlement density** | **#/km^2^** | **10.4** |
| **Roaddensity** | **Road density** | **m/km^2^** | **4.1** |
| **Riverdensity** | **River density** | **m/km^2^** | **0.7** |
| Dem | Elevation | m |  |
| **Veg** | **Vegetation type(categorical)** |  | **4.6** |

The environmental variables in bold, selected by eliminating Pearson’s correlation coefficients with |r| > 0.8, were used in model construction.

Table S2 Analysis of correlation coefficient of the environmental variables

|  | Bi01 | Bi02 | Bi03 | Bi04 | Bi05 | Bi06 | Bi07 | Bi08 | Bi09 | Bi10 | Bi11 | Bi12 | Bi13 | Bi14 | Bi15 | Bi16 | Bi17 | Bi18 | Bi19 | Settledensity | Roaddensity | Riverdensity | Dem |
| --- | --- | --- | --- | --- | --- | --- | --- | --- | --- | --- | --- | --- | --- | --- | --- | --- | --- | --- | --- | --- | --- | --- | --- |
| Bi02 | 0.84 |  |  |  |  |  |  |  |  |  |  |  |  |  |  |  |  |  |  |  |  |  |  |
| Bi03 | 0.82 | 0.97 |  |  |  |  |  |  |  |  |  |  |  |  |  |  |  |  |  |  |  |  |  |
| Bi04 | 0.87 | 0.98 | 0.94 |  |  |  |  |  |  |  |  |  |  |  |  |  |  |  |  |  |  |  |  |
| Bi05 | 1.00 | 0.88 | 0.86 | 0.91 |  |  |  |  |  |  |  |  |  |  |  |  |  |  |  |  |  |  |  |
| Bi06 | 0.97 | 0.69 | 0.68 | 0.74 | 0.95 |  |  |  |  |  |  |  |  |  |  |  |  |  |  |  |  |  |  |
| Bi07 | 0.84 | 1.00 | 0.96 | 0.99 | 0.88 | 0.69 |  |  |  |  |  |  |  |  |  |  |  |  |  |  |  |  |  |
| Bi08 | 0.99 | 0.83 | 0.81 | 0.88 | 0.99 | 0.96 | 0.84 |  |  |  |  |  |  |  |  |  |  |  |  |  |  |  |  |
| Bi09 | 1.00 | 0.80 | 0.78 | 0.83 | 0.99 | 0.99 | 0.80 | 0.99 |  |  |  |  |  |  |  |  |  |  |  |  |  |  |  |
| Bi10 | 1.00 | 0.87 | 0.85 | 0.90 | 1.00 | 0.96 | 0.87 | 0.99 | 0.99 |  |  |  |  |  |  |  |  |  |  |  |  |  |  |
| Bi11 | 1.00 | 0.80 | 0.78 | 0.83 | 0.99 | 0.99 | 0.80 | 0.99 | 1.00 | 0.99 |  |  |  |  |  |  |  |  |  |  |  |  |  |
| Bi12 | -0.94 | -0.95 | -0.93 | -0.95 | -0.96 | -0.85 | -0.95 | -0.93 | -0.92 | -0.96 | -0.92 |  |  |  |  |  |  |  |  |  |  |  |  |
| Bi13 | -0.92 | -0.91 | -0.90 | -0.88 | -0.94 | -0.85 | -0.89 | -0.90 | -0.91 | -0.93 | -0.91 | 0.98 |  |  |  |  |  |  |  |  |  |  |  |
| Bi14 | -0.77 | -0.84 | -0.85 | -0.77 | -0.80 | -0.69 | -0.82 | -0.73 | -0.76 | -0.79 | -0.76 | 0.90 | 0.94 |  |  |  |  |  |  |  |  |  |  |
| Bi15 | 0.28 | 0.51 | 0.52 | 0.39 | 0.32 | 0.18 | 0.47 | 0.23 | 0.26 | 0.30 | 0.26 | -0.50 | -0.55 | -0.74 |  |  |  |  |  |  |  |  |  |
| Bi16 | -0.97 | -0.91 | -0.89 | -0.92 | -0.98 | -0.91 | -0.91 | -0.96 | -0.96 | -0.98 | -0.96 | 0.99 | 0.98 | 0.87 | -0.42 |  |  |  |  |  |  |  |  |
| Bi17 | -0.63 | -0.77 | -0.78 | -0.65 | -0.66 | -0.54 | -0.73 | -0.58 | -0.61 | -0.65 | -0.61 | 0.79 | 0.85 | 0.96 | -0.80 | 0.74 |  |  |  |  |  |  |  |
| Bi18 | -0.95 | -0.91 | -0.90 | -0.90 | -0.97 | -0.89 | -0.90 | -0.94 | -0.94 | -0.96 | -0.94 | 0.99 | 0.99 | 0.91 | -0.50 | 0.99 | 0.80 |  |  |  |  |  |  |
| Bi19 | -0.63 | -0.77 | -0.78 | -0.65 | -0.66 | -0.54 | -0.73 | -0.58 | -0.61 | -0.65 | -0.61 | 0.79 | 0.85 | 0.96 | -0.80 | 0.74 | 1.00 | 0.80 |  |  |  |  |  |
| Settledensity | 0.62 | 0.36 | 0.36 | 0.43 | 0.59 | 0.66 | 0.38 | 0.63 | 0.63 | 0.60 | 0.63 | -0.47 | -0.46 | -0.28 | -0.10 | -0.54 | -0.16 | -0.50 | -0.16 |  |  |  |  |
| Roaddensity | 0.43 | 0.27 | 0.25 | 0.33 | 0.42 | 0.45 | 0.29 | 0.45 | 0.44 | 0.42 | 0.44 | -0.36 | -0.33 | -0.21 | 0.02 | -0.39 | -0.09 | -0.37 | -0.09 | 0.55 |  |  |  |
| Riverdensity | 0.36 | 0.35 | 0.34 | 0.35 | 0.37 | 0.33 | 0.35 | 0.36 | 0.35 | 0.36 | 0.35 | -0.38 | -0.38 | -0.34 | 0.19 | -0.38 | -0.29 | -0.38 | -0.29 | 0.10 | 0.24 |  |  |
| Dem | -0.90 | -0.81 | -0.79 | -0.85 | -0.90 | -0.84 | -0.82 | -0.90 | -0.88 | -0.90 | -0.88 | 0.87 | 0.84 | 0.71 | -0.28 | 0.89 | 0.58 | 0.87 | 0.58 | -0.59 | -0.44 | -0.37 |  |
| Veg | 0.20 | 0.20 | 0.19 | 0.18 | 0.20 | 0.19 | 0.19 | 0.20 | 0.20 | 0.20 | 0.20 | -0.23 | -0.24 | -0.25 | 0.21 | -0.22 | -0.23 | -0.23 | -0.23 | 0.11 | 0.17 | 0.13 | -0.19 |


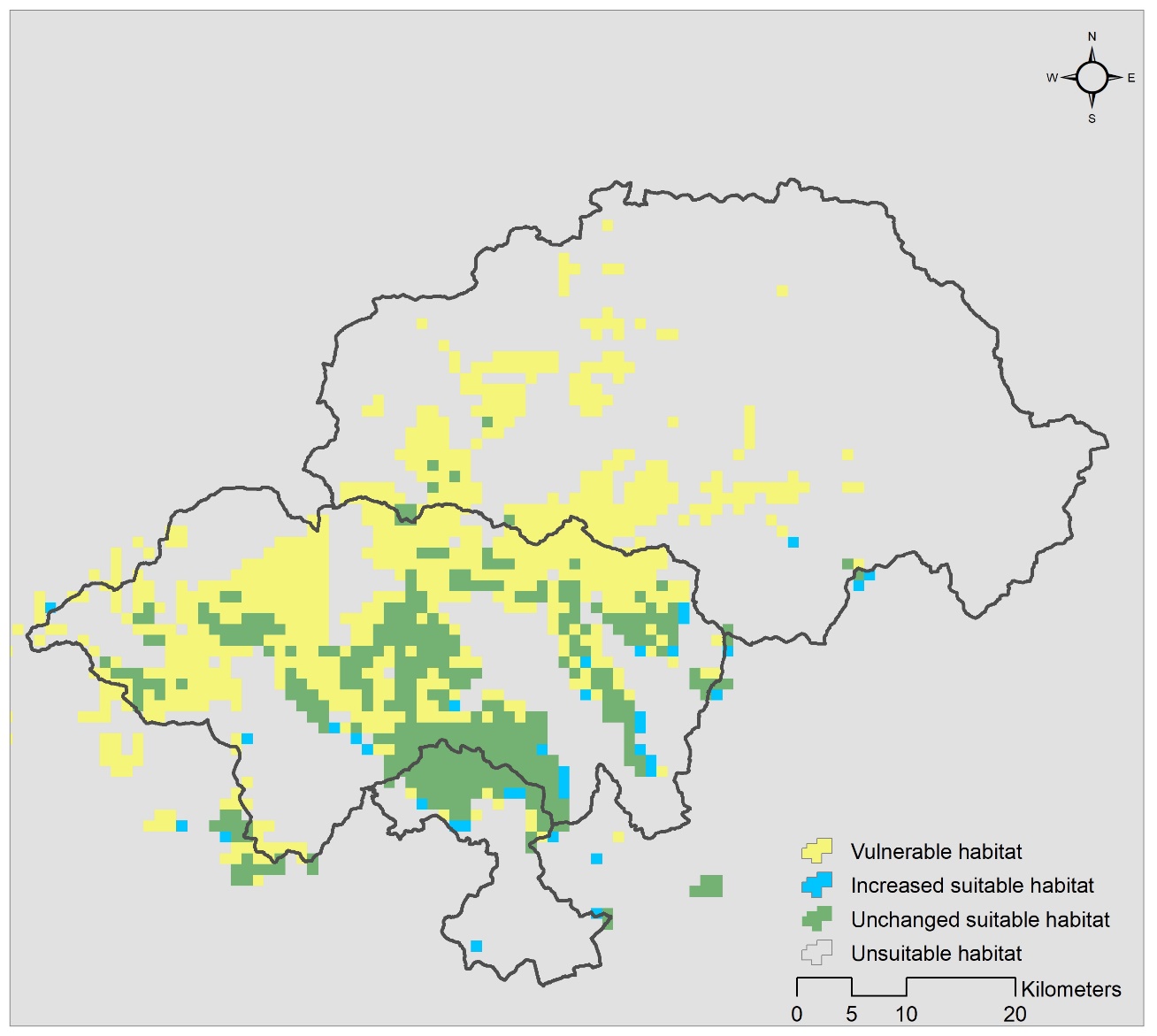


Figure S1.3 Habitat vulnerability of *R. roxellana*
